# Expression of Membrane Bound O-Acyltransferase Domain Containing 7 after Myocardial Infarction and its Role in Lipid Metabolism *in vitro*

**DOI:** 10.7150/ijms.70614

**Published:** 2022-03-14

**Authors:** Xiangdong Li, Zhiyuan Wang, Heyu Meng, Fanbo Meng, Ping Yang

**Affiliations:** 1 Cardiovascular Department of China-Japan Union Hospital of Jilin University.; 2 Ultrasound Department of China-Japan Union Hospital of Jilin University.

**Keywords:** Myocardial Infarction, MBOAT7, Marker, Lipid Metabolism

## Abstract

**Background:** Previous microarray analysis on peripheral blood leukocytes from three patients with acute myocardial infarction (AMI) showed that elevated expression of membrane bound o-acyltransferase domain containing 7(*MBOAT7*) relative to control. To further verify these findings, we investigated more patients and explored the possible mechanisms *in vitro*.

**Objective:** To study alterations in *MBOAT7* expression in leukocytes after AMI, and to explore the relationship between *MBOAT7* and lipid metabolism pathways in hepatocytes *in vitro*.

**Methods:** Ninety patients with AMI and 90 controls were recruited from the Han population in Northeast China. RT-fluorescent PCR was used to measure *MBOAT7* mRNA levels. *MBOAT7* interference and overexpression vectors were constructed and transfected into L-02 hepatocytes and expression was examined by RT-qPCR and western blotting. The expression of *SCAP, LDLR, HMGCR, ACAT1, ABCA1, SREBP1, ACC, FAS, SCD,* and *PPARγ* in the lipid metabolism pathway were investigated by RT-qPCR. Triglyceride and cholesterol levels were measured by ELISA.

**Results:** It was found that *MBOAT7* mRNA levels were elevated in the leukocytes of patients with AMI. Hepatocytes were successfully transfected, shown by attenuated *MBOAT7* mRNA levels in the silenced group (0.41±0.04 vs 1.01±0.07 for control, P=0.0019 <0.01) and raised levels in the overexpressing cells (23.29±0.39 vs 1.00±0.06 for control, P <0.0001). These results were confirmed by western blotting. Expression of the lipid metabolism-related genes was altered in response to *MBOAT7* expression. Triglyceride levels increased after *MBOAT7* silencing (118.40 ± 2.26 vs 70.54 ± 0.25 for control, P<0.0001), as did those of cholesterol (628.30 ± 8.89 vs 544.70 ± 11.04, P = 0.0041) but were not altered on *MBOAT7* overexpression.

**Conclusion:** *MBOAT7* did not affect the metabolism of triglycerides in hepatocytes through fatty acid synthesis and decomposition pathways. The *MBOAT7* level in the peripheral blood can be used as a marker for acute myocardial infarction but cannot be used as a single therapeutic target to regulate lipid metabolism.

## Introduction

According to WHO statistics, cardiovascular disease accounted for 17.9 million deaths in 2016, approximately 32% of all deaths. In 2015 alone, more than 15.9 million people worldwide suffered acute myocardial infarction (AMI) 1. There are many risk factors for AMI, including age, smoking, hypertension, diabetes mellitus, dyslipidemia, and inflammation. Genetic susceptibility also plays a major role. Studying the expression of specific genes in patients with AMI can identify high-risk groups, improve the risk warning of the disease, and provide new diagnostic and treatment plans.

*MBOAT7* encodes a membrane-associated lysophosphatidylinositol acyltransferase, that is specific for arachidonic-CoA as an acyl donor 2-4. The protein reacylates phospholipids in the Land Cycle phospholipid remodeling pathway 2-4. Related metabolic pathways include those related to metabolism and glycerophospholipid biosynthesis. GWAS studies have linked variants of patatin-like phospholipase domain-containing protein 3 (*PNPLA3*), transmembrane 6 superfamily 2 (T*M6SF2*), and *MBOAT7* with all histological stages of Non-alcoholic fatty liver disease (NAFLD) 5, 6. The rs738409 G-allele in *PNPLA3* and the rs58542926 T-allele in *TM6SF2*, however, offered protection against coronary artery disease. The *MBOAT7* rs641738 (T) allele can reduce *MBOAT7* expression in the liver, increasing both susceptibility to and the severity of NAFLD 5, 7-10. Damage caused by NAFLD not only affects the liver and related systems but may also lead to increased risk of type 2 diabetes and cardiovascular and chronic kidney disease 11. Indeed, cardiovascular disease has been found to be the principal cause of death in NAFLD patients, followed by liver-related diseases 12-14. We hypothesized that MBOAT7 may be a key link between NAFLD and coronary heart disease, and investigated both changes in MBOAT7 expression in peripheral blood leukocytes after AMI and the possible molecular mechanisms in hepatocytes.

## Methods

### Admission and exclusion criteria

Ninety inpatients with a clear diagnosis of AMI were enrolled at the Department of Cardiology, China-Japan Union Hospital of Jilin University from January 2016 to April 2016. All patients were from the Han population in northern China. The diagnostic criteria were based on the globally accepted definition of AMI announced in 2012 15, meeting the criterion of confirmation by coronary angiography of at least 70% stenosis in the main coronary arteries (left main, descending artery, circumflex, and right coronary arteries) and at least one of their branches. The control group consisted of 90 patients with non-coronary atherosclerotic heart disease where the stenosis in the main coronary arteries and branches was less than 50%. All patients were tested for fasting blood glucose and lipids in the morning after admission. Each patient signed an informed consent form, and the study was approved by the Ethics Committee of China-Japan Union Hospital. The basic clinical information of the patients is shown in Table 2.

### Sample collection and processing

Four milliliters of fasting venous blood were drawn from study participants two days after admission, added to an EDTA anticoagulation tube, and stored at 4 °C. Follow-up experiments were performed within two hours of sample collection. A total RNA extraction kit (RNAsimple Total RNA Kit, Tiangen Biochemical Technology Co., Ltd., Beijing) was used to extract RNA from the leukocytes, according to the instructions, and the quality and concentrations of the RNA were determined using 1.5% agarose electrophoresis and spectrophotometry (Nanodrop 2000), respectively. cDNA was synthesized from 1 µg of RNA using the Revertra Ace qPCR RT kit (TOYOBO, Shanghai), and stored at -20 °C for the next step of RT fluorescence quantification PCR.

### qPCR detection of gene expression level

cDNA samples were diluted 10 times and amplified using the SYBR fluorescence quantitative kit (SYBR Premix Ex Taq TM, TaKaRa, Dalian). The 20 µl reaction system included 10 µl SYBR Premix Ex Taq TM, 0.5 µl primers (upstream and downstream, 10 µmol/L), 8 µl nuclease-free double-distilled water, and 1 µl cDNA template. An Mx3005P real-time fluorescent quantitative PCR system (StrataGene) was used for amplification. The reaction conditions were 95 °C for 10 minutes, 95 °C for 15 seconds, 60 °C for 20 seconds, 72 °C for 20 seconds, 95 °C for 1 minute, 55 °C for 30 seconds, and 95 °C for 30 seconds, for 40 cycles. *GAPDH* was used as the internal reference, and three replicates of each sample were used. The cycle threshold (Ct) obtained for each sample was expressed as relative expression 2^-ΔCT^ (ΔCT=target gene CT-internal reference gene CT). The relative expression of the AMI group versus the control group was statistically analyzed by the 2^-ΔΔCt^ method.

### Overexpression and interference vector construction

The overexpression vector, PgCMV/EGFP/Neo-MBOAT7, was designed according to the human *MBOAT7* gene sequence published on the NCBI website, and the negative control was the PgCMV/EGFP/Neo empty vector. The interference vector was pGPU6/GFP/Neo-MBOAT7, and the negative control was pGPU6/GFP /Neo-shNC. All the vectors were synthesized by the Shanghai Gima Biological Company.

### Cell culture and transfection

Human hepatocytes (L-02, Shanghai Zhongqiao Xinzhou Biotechnology Co.) were cultured in a constant temperature 5% CO_2_ incubator at 37 °C. The medium was RPMI 1640 with 10% fetal bovine serum and 100 U/ml penicillin/streptomycin. When the cells had reached 70%-80% confluence, they were subcultured by trypsinization into 6-well plates (2×10^5^/ml). The cells were cultured for 24 hours and when 70% confluent, were transfected. Four 1.5-ml sterile centrifuge tubes, filled with 150 μl serum- and antibiotic-free medium were prepared, and 3 μg pBI-CMV3-MBOAT7 overexpression vector, pBI-CMV3 empty vector, 3 µg pGPU6/GFP/Neo-MBOAT7 interference vector, or pGPU6/ For GFP/Neo-shNC negative control vector were added to the tubes. After mixing the plasmid and the medium thoroughly, 7.5 μl of the transfection reagent (FuGENE®HD LLC, USA) was added to each tube. The mixture was stirred with a pipette tip and allowed to stand for 15 minutes. Antibiotic-free medium was added to the tissue culture plate and the cell-plasmid mixture was added to the plate evenly in a clockwise direction, with gentle shaking to ensure even distribution. The plates were incubated for 24 hours, and the cells and the GFP distribution were observed under a fluorescence microscope (Thermo Fisher, Waltham, MA, USA).

### Detection of *MBOAT7* expression at the mRNA and protein levels

RNA and cDNA were obtained from cells 24 hours after transfection, as described above. The cDNA was used as a template for fluorescent quantitative PCR. The 20 µl reaction system contained 10 µl SYBR® PremixEx taqTM, 0.4 μl primer, 1 μl cDNA, and 8.2 μl water. The amplification conditions were 95 °C for 10 minutes; 95 °C for 10 seconds, 60 °C for 30 seconds, 72 °C for 15 seconds and 35 cycles, and 55 °C for 15 seconds. Fluorescent qPCR amplification of *MBOAT7* and *GAPDH* (internal reference) was performed using different template cDNAs, with three replicates per sample to ensure accuracy.

The total protein was extracted from the cells 48 hours after transfection using RIPA lysis buffer (Beyotime Biotechnology, China), and the protein concentration was measured by the BCA Protein Assay kit (Beyotime Biotechnology). The protein concentrations of different samples were adjusted to ensure that the same amount of protein was loaded. After separation of the proteins on SDS-PAGE, the proteins were transferred to PVDF membranes and probed with the appropriate antibodies.

### Investigation of lipid-associated gene expression

A total of 10 pairs of RT-qPCR primers for lipid metabolism-related genes were synthesized. The primer list is shown in Table 1. Using different cDNA templates and *GAPDH* as the internal reference, we investigated the levels of triglyceride-associated genes, including: sterol regulatory element binding protein* (SREBP1),* acetyl-coenzyme A carboxylase* (ACC),* fatty acid synthase *(FAS),* stearyl carboxylase A dehydrogenase *(SCD1),* and peroxisome proliferators-activated receptors γ *(PPARγ)*, as well as the expression of cholesterol metabolism-related genes, including: SREBP Cleavage-Activating Protein *(SCAP).* Low density lipoprotein receptor *(LDLR),* 3-hydrox-3-methylglutaryl-CoA reductase *(HMGCR),* acetyl-CoA acetyltransferase 1 *(ACAT1),* and ATP binding cassette subfamily A member 1 *(ABCA1)*. The thermal cycling conditions were the same as before. There were also three replicates for each sample to ensure the accuracy of the test.

### Measurement of triglyceride and cholesterol

The triglyceride and cholesterol concentrations in hepatocytes were measured 48 hours after transfection using the cell triglyceride and cholesterol assay kit (Applygen Technologies, Beijing, China), according to instructions.

Firstly, after removal of spent medium, the cells were washed three times with PBS and 300 μl of lysate (the amount of lysate added depending on the cell density) were added and allowed to stand for 10 minutes. The cells were collected, mixed, centrifuged, and the supernatant was used for protein quantification. The supernatant was warmed to 70 °C, centrifuged at 2000 rpm for 5 minutes, and the retaining the supernatant. Fifty microliters of the supernatant were added each well of an ELISA plate, using a blank control group and three replicates of each sample. After addition of 150 μl of working solution and allowing to react for 10 minutes at 37 °C, the absorbances at 550 nm were measured using a microplate reader (Infinite 200 PRO multi-function microplate reader, Tecan, Switzerland), and the triglyceride and cholesterol concentrations were calculated using the standard curve and expressed in terms of the protein concentrations.

### Statistical analysis

SPSS software (IBM Corp., Armonk, NY, USA), version 22.0 was used to analyze the data and GraphPad Prism version 7.0 was used for the figures. Normally distributed data were represented by means ± standard deviation (SD). Independent-samples t-tests were used to compare differences between groups. P-values below 0.05 was considered statistically significant.

## Results

The two patient groups did not differ significantly in terms of age, sex, BMI, diabetes, hypertension, smoking, drinking, and comorbidities (Table 2).

There were differences in fasting blood glucose and high-density lipoprotein (HDL) cholesterol values between the two groups. Specifically, glucose levels were higher in the AMI patients, while the HDL cholesterol levels were lower than controls. Total cholesterol, plasma triglyceride, and LDL cholesterol values did not differ significantly.

### *MBOAT7* expression

**Figure 1:**
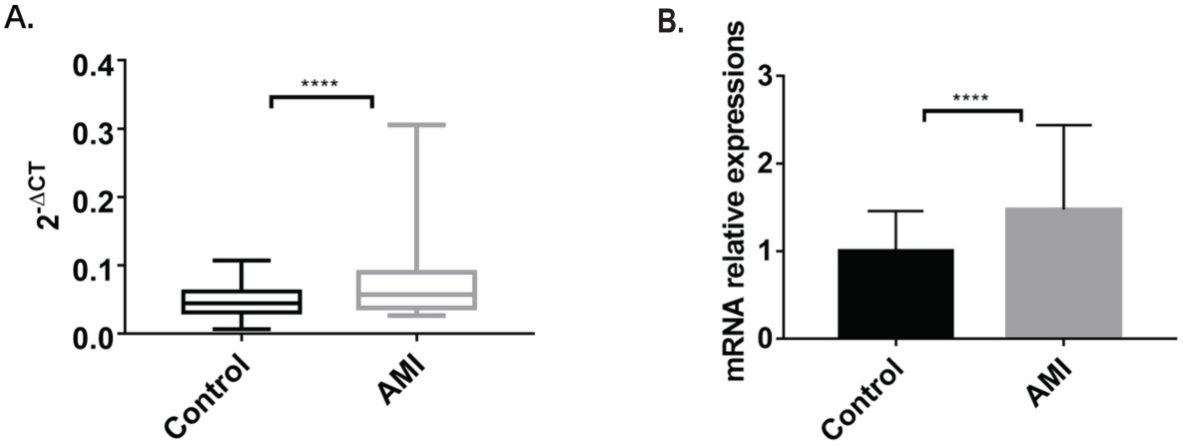
The comparison of MBOAT7 expression between AMI and control group. **(A)** MBOAT7 mRNA expression Control vs AMI. **(B)** MBOAT7 mRNA relative expression Control vs AMI. AMI=acute myocardial infarction. ****P<0.0001.

The RT fluorescence qPCR results showed a single melting peak for *MBOAT7,* together with high specificity and an amplification curve of a significant “S” type. The PCR products were visible as a single bright band, at about 93 bp, in agarose gel electrophoresis, which was consistent with the expected fragment size. The ΔCT values of the samples were the means of three replicates, and the standard deviation was within the required range. The 2^-ΔCT^ of the case group was higher (0.07096±0.004887) than that of the control group (0.04816±0.00231) (P<0.0001). Relative expression of *MBOAT7* between the groups was determined by 2^-ΔΔCt^. This indicated that *MBOAT7* levels in leukocytes from AMI patients were higher, with a relative expression of 1.473 ± 0.1015 vs 0.9999 ± 0.04796 for the control (P<0.0001), as shown in Figure 1.

### *MBOAT7* mRNA and protein after transfection

**Figure 2:**
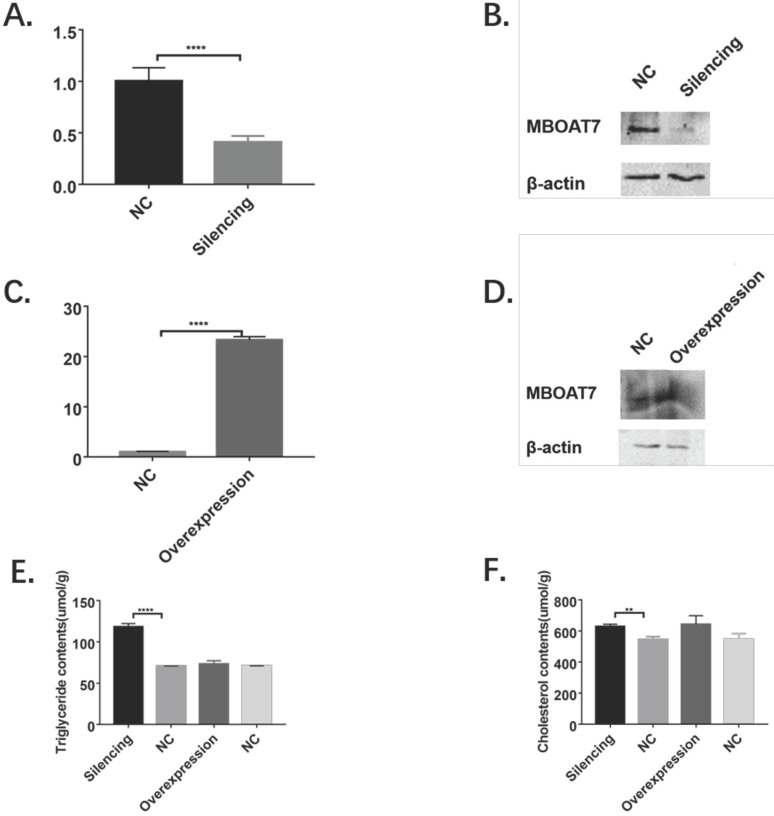
Alterations of MBOAT7 mRNA and protein levels and effects on triglyceride and cholesterol after MBOAT7 knockdown and overexpression in L-02 cells. (**A**). MBOAT7 mRNA relative expression after MBOAT7 knockdown Control vs Silencing. **(B)** Western Blot for MBOAT7 after MBOAT7 knockdown Control vs Silencing. **(C)** MBOAT7 mRNA relative expression after MBOAT7 overexpression Control vs Overexpression. **(D)** Western Blot for MBOAT7 after MBOAT7 overexpression Control vs Overexpression. (**E**). Changes in triglyceride content after MBOAT7 knockdown and overexpression (umol/g). **(F)** Changes in cholesterol content after MBOAT7 knockdown and overexpression (umol/g). **P<0.01, ****P<0.0001.

Significantly reduced levels of MBOAT7 mRNA were observed in cells transfected with the interference plasmid (0.41±0.04 vs 1.01±0.07 for control, P=0.0019 <0.01), while the mRNA level in the overexpressing cells was raised (23.29±0.39 vs 1.00±0.06 for control, P<0.01), as shown in Figure 2.

The MBOAT7 protein levels were determined 48 hours after transfection, using β-actin as reference. Western blots showed significantly reduced MBOAT7 levels in the interference group and a corresponding elevation in the overexpressing cells (Figure 2).

### Changes in cholesterol and triglyceride metabolism-related gene levels

**Figure 3:**
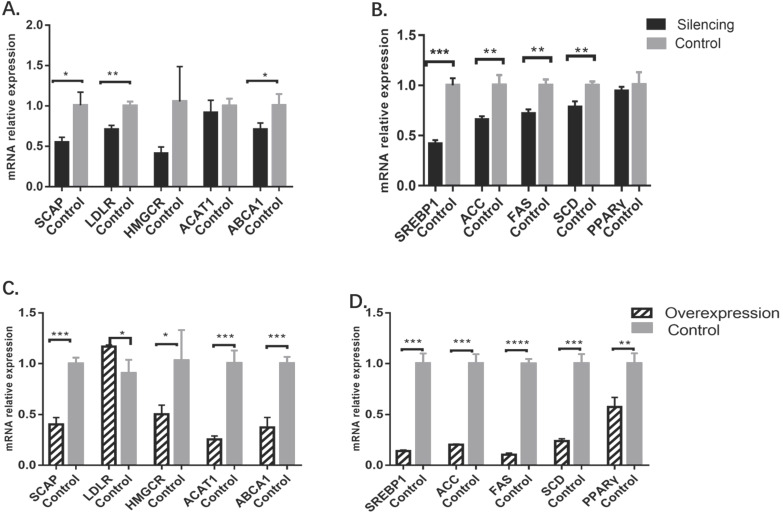
** Changes in cholesterol and triglyceride metabolism-related gene levels after MBOAT7 knockout and overexpression. (A)** Changes in cholesterol metabolism-related gene levels after MBOAT7 Silencing. **(B)** Changes in triglyceride metabolism-related gene levels after MBOAT7 Silencing. **(C)** Changes in cholesterol metabolism-related gene levels after MBOAT7 Overexpression. **(D)** Changes in triglyceride metabolism-related gene levels after MBOAT7 Overexpression. *P<0.05, **P<0.01, ***P<0.001, ****P<0.0001.

In the *MBOAT7*-knockdown cells, the relative expression of the cholesterol-related gene *SCAP* was 0.55±0.03 vs 1.01±0.09 for the control (P=0.0101), while that of *LDLR* was 0.71±0.03 vs. 1.00±0.03 for the control (P=0.0022), and *ABCA1* was 0.71±0.04 vs 1.01±0.08 for the control (P=0.0331). Expression of *HMGCR* and *ACAT1* were essentially the same between the two groups. For genes related to triglyceride metabolism, it was found that the relative expression of *SREBP1* was 0.42±0.02 vs 1.00±0.04 for the control (P= 0.0002), while that of *ACC* was 0.66±0.02 vs 1.00±0.06 (P= 0.0047), that of *FAS* was 0.72 ± 0.03 vs 1.00 ± 0.03 (P = 0.0025), and that of *SCD* was 0.78 ± 0.03 vs 1.00 ± 0.02 (P = 0.0055). The levels of *PPARγ* showed no change (Figure 3).

In MBOAT7-overexpressing cells, the relative expression of *SCAP* was 0.40 ± 0.04 vs 1.00 ± 0.03 (P=0.0003), and that of *LDLR* was 1.17 ± 0.01 vs 0.91±0.08 (P=0.0264). *HMGCR* expression was also raised 0.51±0.05 vs 1.03±0.17 (P=0.0427), as were the expression of *ACAT1* (0.26 ± 0.02vs1.01±0.07; P= 0.0005), *ABCA1* (0.37 ± 0.06 vs 1.00 ± 0.04; P = 0.0008), *SREBP1* (0.14 ± 0.01 vs 1.00 ± 0.06; P = 0.0001), *ACC* (0.20 ± 0.01 vs 1.00 ± 0.05; P = 0.0001), FAS (0.11 ± 0.01 vs 1.00 ± 0.03; P<0.0001), *SCD* (0.24 ± 0.01 vs 1.00 ± 0.05; P= 0.0001), and PPARγ (0.58 ± 0.05 vs 1.00 ± 0.06; P=0.0055) (Figure 3).

### Changes in intracellular triglyceride and cholesterol content

In the knockdown cells, the triglyceride content was found to be significantly increased (118.40 ± 2.26 vs 70.54 ± 0.25, P <0.0001), as was the cholesterol content (628.30 ± 8.89 vs 544.70 ± 11.04, P = 0.0041), as shown in Figure 2.

In the *MBOAT7*-overexpressing cells, no differences in either triglyceride (73.02 ± 2.46 vs 70.76 ± 0.19 for the control, P=0.41) or cholesterol concentrations (642.10 ± 32.41 vs 545.80 ± 21.22 for the control, P=0.068) were observed (Figure 2).

## Discussion

In a previous study by our research team, microarray analysis of mRNAs in the peripheral blood leukocytes of three AMI patients identified 559 differentially expressed genes. Of these, 288 were upregulated and 271 were downregulated 16. These genes were associated with 128 cellular components and were involved in 521 biological processes and 151 molecular functions. KEGG pathway analysis indicated that the differentially expressed genes were involved in 107 pathways, including “systemic lupus erythematosus”, “lipid metabolism”, “Alzheimer's disease”, “receptor interaction”, “apoptosis”, “MAPK signaling pathway”, and “insulin signaling pathway”. Yang et al. 17-20 analyzed *ACSL1, PRMT5*, *PIK3C2A*, and *CPNE3* levels in additional patients, finding that the results agreed with the findings of the microarray, confirming the reliability of the microarray results and the feasibility of single-gene diagnosis of diseases. The microarray results showed elevated *MBOAT7* expression after AMI 16. Here, we evaluated the expression of *MBOAT7* in more patients and found that it was markedly higher in AMI patients, consistent with the microarray observations.

The *MBOAT7* rs641738 (T) gene polymorphism can lead to reduced *MBOAT7* transcription in hepatocytes, increasing both the susceptibility to and severity of NAFLD 5, 7-10. Another study confirmed the *MBOAT7* hepatocyte expression findings while showing raised *MBOAT7* mRNA levels in peripheral blood leukocytes where it appears to have an anti-inflammatory effect 21. In this study, the white blood cells in the acute myocardial infarction group were significantly increased, which is consistent with the previous research conclusions, suggesting that the body is in an obvious inflammatory process after acute myocardial infarction. After the release of inflammatory factors into the blood, the body responds by starting an anti-inflammatory process, including increasing the expression of MBOAT7 gene, thereby reducing the damage caused by inflammation to the body. Further investigation into the functions of *MBOAT7* and its possible role in inflammation is needed.

Here, the comparison between the AMI and control groups showed alterations in the fasting blood glucose and HDL lipoprotein levels. Glucose levels in the AMI groups were higher, while HDL levels were lower than in controls. The total cholesterol, triglyceride, and LDL levels remained unchanged. The findings of increased blood sugar and decreased HDL levels are consistent with the documented characteristics of the AMI population. However, we did not see differences in the cholesterol and triglyceride levels between the two groups, which is contrary to current research findings, especially the LDL cholesterol which is the main indicator of current lipid-lowering therapy and is a well-documented risk factor for cardiovascular disease. However, it is possible that this result may not necessarily reflect the overall situation due to the limited number of patients investigated. In addition, many patients in the AMI group had received statin treatment before admission, so the current results reflect the serum cholesterol level after lipid-lowering therapy, and may not truly reflect the relationship between cholesterol levels and the risk of AMI.

The *in vitro* experiments showed that, after knockdown of MBOAT7 expression, the concentrations of cholesterol and triglycerides in the hepatocytes were significantly elevated in comparison with the controls, while not altering significantly on *MBOAT7* overexpression. It has previously been reported that the *MBOAT7* rs641738 (T) allele attenuates MBOAT7 expression in hepatocytes together with increased lipid levels in the cells 9, 22. This is consistent with our *in vitro* results.

SREBP consists of three isoforms, of which SREBP-1c has been reported to be involved in fatty acid and insulin-related glucose metabolism 23. Raised SREPB-1c levels in the livers of dairy cows have been linked to lipid accumulation in the organ, and SREBP-1c levels correspond to triglyceride accumulation in the liver 24, 25. ACC1, FAS, and SCD1 are lipid synthesis-associated enzymes and are targeted by SREBP-1c 26. ACC1 catalyzes the acetyl-CoA/malonyl-CoA conversion during fatty acid synthesis 27. FAS is a regulator of fatty acid synthesis, and SCD1 is responsible for the desaturation and extension of fatty acids 28, 29. Thus, these genes are linked to fatty acid synthetic and metabolic processes. Our study found that after interfering with *MBOAT7* expression, the triglyceride content of hepatocytes increased markedly. qPCR analysis demonstrated significant attenuation of *SREBP1, ACC, FAS*, and *SCD* expression, while that of *PPARγ,* responsible for the oxidative decomposition of fatty acids, remained unchanged. This suggests that *MBOAT7* does not cause the increase in triglycerides through fatty acid synthesis and decomposition. A recent report confirmed that MBOAT7 can promote triglyceride synthesis in hepatocytes by increasing the conversion of phosphatidylinositol 30. This may be the principal mechanism behind the action of the *MBOAT7* rs641738 (T) allele causing and aggravating NAFLD. After *MBOAT7* overexpression, there was no significant change in the triglyceride content of hepatocytes. qPCR analysis indicated downregulation of several fatty acid synthesis-associated genes together with that of *PPARγ*. Downregulation of *PPARγ* can lead to a decrease in the oxidation and decomposition of triglycerides. However, there were also lowered levels of genes related to synthesis and decomposition, with the final result that there was no significant change in the triglyceride content of the cell. This also showed that simply increasing the expression of *MBOAT7* cannot reduce the triglyceride content.

*MBOAT7* knockdown resulted in increased cholesterol levels in hepatocytes. Previous investigations have mostly emphasized the influence of *MBOAT7* on triglyceride metabolism, and there are few studies on cholesterol. Thangapandi VR et al constructed *Mboat7* knockout mice and found that the mice developed spontaneous steatosis, the main feature of which was a raised cholesterol ester level in the liver after 10 weeks 31. Marcin Krawczyk et al. analyzed 84 obese patients who had undergone bariatric surgery, observing that not only was the *MBOAT7* rs641738 (T) allele associated with elevated triglyceride levels, but was also linked to raised levels of total cholesterol, LDL, and serum glucose 32. The changes in cholesterol observed in these studies are consistent with our findings. In our study, the cholesterol metabolism pathway analysis found that the expression of LDLR decreased after *MBOAT7* knockdown and increased after *MBOAT7* overexpression, while the expression of *ACAT1* did not change significantly after MBOAT7 knockdown but was significantly reduced on overexpression. The expression of other genes in the pathway such as *SCAP, HMGCR,* and *ABCA1* were all downregulated after *MBOAT7* knockdown or overexpression. LDLR is the main receptor on peripheral cells for cholesterol uptake from the circulation, which is efficiently removed by LDLR endocytosis 33. *ACAT1* regulates cholesterol esterification which can prevent the accumulation of free cholesterol within cells, regulate the storage or secretion of cholesterol, and maintain the balance between free cholesterol and cholesterol esters 34, 35. Since the cholesterol concentrations we detected include the sum of free cholesterol and cholesterol ester content, the change in *ACAT1* will theoretically not affect the measured cholesterol content. In this study, after the expression of MBOAT7 gene was changed, the content of total cholesterol changed. So, the changes in ACAT1 were the result, not the cause. After MBOAT7 gene knockout, the expression of LDLR was decreased, and the content of cholesterol was increased too. After MBOAT7 overexpression, the expression of LDLR was increased, but there was no significant change in cholesterol. LDLR showed opposite changes, but the total cholesterol content did not have the opposite trend. In addition, the decreased expression of LDLR in hepatocytes would lead to a decrease in cholesterol uptake by endocytosis, which is inconsistent with the result of the decreased LDLR expression and increased cholesterol content in this study. This indicated that the knockout and overexpression of MBOAT7 resulted in different trends in LDLR, but not the reason for the change in cholesterol content. The possible reason is that the MBOAT7 gene causes changes in cholesterol content through other mechanisms, and negative feedback acts on LDLR, resulting in changes in the expression of LDLR.

## Conclusion

This study found that *MBOAT7* was highly expressed in the peripheral blood leukocytes of AMI patients. Knockdown of *MBOAT7* resulted in raised levels of intracellular triglycerides and cholesterol. These changes do not appear to involve fatty acid synthesis and catabolism pathways. Overexpression of MBOAT7 did not affect the triglyceride and cholesterol levels in the cells. This study suggests that *MBOAT7* can be used as a peripheral blood marker after AMI but cannot be used as a single therapeutic target to regulate lipid metabolism.

## Figures and Tables

**Table 1:** Primer list

Gene Symbol	Species	Sequence (5' -> 3')
MBOAT7	Human	TCCGCAACATCGACTGCTAC
		CGCAGGACATAGGAACGGG
SREBP1^36^	Human	CGGAACCATCTTGGCAACAGT
		CGCTTCTCAATGGCGTTGT
SCD^36^	Human	TTCCTACCTGCAAGTTCTACACC
		CCGAGCTTTGTAAGAGCGGT
ACC^36^	Human	ATGTCTGGCTTGCACCTAGTA
		CCCCAAAGCGAGTAACAAATTCT
PPARγ^36^	Human	ACCAAAGTGCAATCAAAGTGGA
		ATGAGGGAGTTGGAAGGCTCT
FAS^36^	Human	AAGGACCTGTCTAGGTTTGATGC
		TGGCTTCATAGGTGACTTCCA
HMGCR^37^	Human	TACCATGTCAGGGGTACGTC
		CAAGCCTAGAGACATAATCATC
LDLR^37^	Human	CAATGTCTCACCAAGCTCTG
		TCTGTCTCGAGGGGTAGCTG
ACAT1^38^	Human	AACACCCATTGGATCTTTTTTAGG
		TGCAATGGAACCAAGCTTAGTG
ABCA1^39^	Human	GGGGTGGTGTTCTTCCTCATTAC
		CCGCCTCACATCTTCATCTTC
SCAP	Human	CCGCCTCACATCTTCATCTTC
		GGGGCGAGTAATCCTTCACA
GAPDH	Human	ACATCATCCCTGCCTCTACTG ACCACCTGGTGCTCAGTGTA

**Table 2:** Baseline characteristics of control and AMI groups

	Control group	AMI group	P
N	90	90	
Age (years)	57.44±1.28	65.79±1.57	<0.0001
Gender (male/female)	54/36	63/27	0.160
BMI	24.57±0.41	27.08 ± 2.346	0.29
Systolic blood pressure	136.79±21.64	131.31±25.48	0.123
Diastolic blood pressure	82 (75.5,90)	80 (70.5,90)	0.186
Glucose fasting	5.857±0.1864	7.272±0.3457	0.0003
Total Cholesterol	4.464±0.1169	4.572±0.1409	0.5540
Triglycerides	1.675±0.1319	1.799±0.1249	0.4997
LDL-C	2.897±0.08641	3.11±0.1124	0.1337
HDL-C	1.07±0.02978	0.9736±0.02485	0.0143
WBC (×10^9^)	6.367±0.2248	8.278±0.333	<0.0001
History of diabetes mellitus	12/78	19/71	0.893
History of hypertension	37/53	37/53	1
History of smoking	39/51	44/46	0.455
History of drinking	11/79	7/83	0.32

## Abbreviations

MBOAT7: membrane bound O-acyltransferase domain containing 7
AMI: acute myocardial infarction
PNPLA3: patatin-like phospholipase domain-containing protein 3
TM6SF2: transmembrane 6 superfamily 2
HDL: high-density lipoprotein
ACSL1: long-chain acyl-coenzyme A synthetases 1
ACC: acetyl-coenzyme A carboxylase
FAS: fatty acid synthase
PPARγ: peroxisome proliferators-activated receptors γ
SREBP1: sterol regulatory element binding protein
SCD1: stearyl carboxylase A dehydrogenase
SCAP: SREBP Cleavage-Activating Protein
LDLR: Low density lipoprotein receptor
HMGCR: 3-hydrox-3-methylglutaryl-CoA reductase
ACAT1: acetyl-CoA acetyltransferase 1
ABCA1: ATP binding cassette subfamily A member 1
